# Cognitive Deficits in Alcoholism: Approaches to Theoretical Modeling

**Published:** 1995

**Authors:** Kathryn G. Ingle, Herbert J. Weingartner

**Affiliations:** Kathryn G. Ingle, M.A., is a science editor of Alcohol Health & Research World, Washington, DC. Herbert J. Weingartner, Ph.D., is chief of the Cognitive Neurosciences Section in the Laboratory of Clinical Studies, National Institute on Alcohol Abuse and Alcoholism, Bethesda, Maryland

**Keywords:** cognitive process, AOD dependence, literature review, AOD impairment, brain imaging, performance test, scientific model, treatment

Over the past 20 years, the field of cognitive neuroscience has progressed toward reaching its primary goal of defining what mechanisms in the brain underlie distinct domains of human cognitive functioning, such as components of memory and attention. Cognitive neuroscientific research has advanced understanding not only of how each domain operates uniquely from the others but also of how separate domains work together. Evidence from cognitive research shows that each of the complex cognitive functions is the product of several separate brain operations. Many studies focus on these operations, examining, for instance, the separate processes the brain may use to store different types of information in memory as opposed to the processes used to retrieve the memory later.

Neurocognitive methods also are being used to search for the particular brain functions that are impaired and spared in alcoholism.^1^ Studies are attempting to answer questions such as the following: What cognitive characteristics may place a person at risk for developing alcoholism? What are the cognitive aspects of addiction? What effects does alcohol have on the brain’s neurophysiology?

Cognitive neuroscience’s research tools, measures, and approaches to the study of normal brain function also have much potential for helping to answer questions about alcohol-related impairments. In a reciprocal manner, and following a research tradition that hails from the mid-19th century, studies of impaired brain function undoubtedly will shed light on the workings of normal human cognition. Clinical studies of people with particular cognitive deficits—including those resulting from alcoholism—in whom other brain functions are spared have been used for over 100 years to help determine how brain functions may be differentiated. This article reviews the use of some of cognitive neuroscience’s tools in studies of alcoholics, such as brain imaging, laboratory procedures for studying cognition (which allows functional analyses of cognitive operations), and drug challenge methods. It also examines the automatic-reflective operations model of cognitive organization that appears useful for defining alcoholics’ impairments. (For further readings on the cognitive neuroscience perspective in research, see Gazzaniga 1988; Jacoby et al. 1992; Lister and Weingartner 1991; Posner 1989; and Schacter 1992.)

## A Foundation: Experimental Psychology

The field of experimental psychology has contributed to cognitive research on alcoholics and other impaired populations by focusing on functional analyses of patients’ behavior without tying in aspects of biological science, such as neurochemistry. Experimental psychologists have systematically compared patterns of impaired functioning, such as those seen in alcoholism, with the cognitive profiles expressed by populations of neuropsychiatric disorder patients. These studies have advanced knowledge of cognitive functioning in alcoholics by examining questions such as the following:

- To what extent do cognitive deficits apparent in the recently detoxified alcoholic disappear with continued abstinence?
- Are the deficits apparent in alcoholics attributable to the same underlying causes as the profound amnesias associated with alcoholic Korsakoff’s syndrome (for a definition of this and other terms in this article, see glossary, pp. 136–137).
- Are the cognitive changes associated with alcoholism similar to or different from the cognitive changes associated with normal aging?^2^
- Are alcoholics’ cognitive impairments associated with changes seen in other neuropsychiatric syndromes, such as depression or anxiety?

Experimental psychology research has formed a foundation for the more recent cognitive neuroscience approaches to studying these and other questions concerning alcoholism’s effects on cognition. (For reviews of cognitive research in experimental psychology, see Brandt et al. 1983; Lister et al. 1987; Oscar-Berman 1980; Oscar-Berman and Ellis 1987; Riege 1987; Salmon and Butters 1987; and Wilkinson and Poulos 1987.)

## Tools of Cognitive Neuroscience

Recent innovations in cognitive neuroscience technology have resulted in many tools useful for examining separate brain operations and for testing specific cognitive domains. These include neurocognitive tests, imaging techniques, and drug challenge research.

### Neurocognitive Tests

Instruments designed to study specific areas of cognitive functioning assist in analyses of alcoholics’ functional performance, that is, how they execute specific behaviors. Alcoholics’ performance may then be compared with that of nonalcoholics to determine whether the alcoholics have impairments in the operations tested, thereby pinpointing the brain operations that contribute to that particular deficit (see the article by Nixon, pp. 97–103).

### Imaging Techniques

Imaging studies provide the neurobiological information to complement neurocognitive tests. Imaging techniques, such as magnetic resonance imaging (MRI) and positron emission tomography (PET), are used to examine anatomy (e.g., whether a certain brain area is larger or smaller in alcoholics than in nonalcoholics); glucose utilization in specific brain areas, which indicates whether these brain areas are less active in alcoholics than in nonalcoholics; and activity in brain systems controlled primarily by one or another neurotransmitter, such as dopamine or serotonin (for further discussion of neurotransmitters, see sidebar by Hiller-Sturmhöfel, p. 128).

Typically, brain imaging data collected from alcoholic subjects as they perform well-defined cognitive tasks focus on a single type of cognitive functioning. The imaging techniques detect the brain areas involved in performing these tasks, and these images are compared with those of nonalcoholics performing the same test to determine whether differences exist in the brain areas involved. In addition, brain images generated while patients complete one task are compared with brain images obtained from the same patients under another test condition, such as while under the influence of a drug that mimics alcohol’s effects. These methods demonstrate how chronic excessive alcohol consumption alters the workings of brain structures.

Coupled with the functional analyses from neurocognitive tests, data from imaging techniques reveal which brain structures are responsible for certain impaired behaviors among alcoholics. For example, Weingartner and colleagues (in press *a*) investigated alcoholics’ performance and their correlated brain glucose utilization when different components of memory were tested. The researchers found that those alcoholics who were unable to refrain from making errors during some memory tasks also had decreased glucose utilization in brain areas associated with cognitive functions under voluntary control (as opposed to those involved in reacting to external stimuli; discussed further below). In contrast, the alcoholics’ glucose metabolism was not decreased in brain areas that appear to control other components of memory. The researchers hypothesized that alcoholics express specific cognitive deficits in functions that require control and reflection, or voluntary recall.

### Drug Challenge Research

Some cognitive deficits displayed by alcoholics may be subtle and are not apparent when the alcoholic is sober. However, when alcoholics are treated with drugs that simulate some of alcohol’s effects, these subtle deficits may be amplified. Thus, whether the subject displays the deficits depends on the presence or absence of a drug. These deficits are termed “state-dependent.” State-dependent learning and memory have been produced in normal (e.g., nondepressive) subjects given drugs that mimic depression or other conditions, including an alcohol-induced state (using benzodiazepines, which mimic the effects of alcohol). When asked about past experiences, subjects under such conditions remember different life events from the ones they recalled when they had not been given any drug. They may remember certain events more readily when they experience the same emotions they felt at the time the event occurred. For example, after taking benzodiazepines, they may more readily remember events associated with alcohol-intoxicated states.

Drug challenge studies, which involve inducing state-dependent changes, reveal not only that alcoholics’ functioning changes in a simulated alcohol-induced state but that the change is qualitatively different from the one that nonalcoholics exhibit under the same conditions. For example, when administered the benzodiazepine Halcion^®^, alcoholics exhibit more difficulty suppressing inappropriate responses from memory (i.e., intrusion errors) than do nonalcoholics given Halcion. Also, alcoholics under the influence of Halcion generate more uncommon, though not inappropriate, words when asked to think of words in a specific category (e.g., types of vegetables). These findings demonstrate a qualitative change in the alcoholics’ thinking that is dependent on their drug-induced state. In their study, Weingartner and colleagues (in press *a*,*b*) conclude that “in the presence of a drug whose acute effects resemble alcohol, otherwise healthy alcoholics demonstrate substantial changes in how they think about commonplace types of stimuli.” Thus, not only do sedative drugs appear to impair some cognitive functions in a manner that models impairments observed in alcoholics, but they also may produce an alteration in the way alcoholics process information, a change that does not occur among nonalcoholics given sedative drugs (Weingartner et al. in press *a*,*b*).

## Modeling Cognitive Processes

Neuroscientific studies attempt to weave data concerning specific cognitive operations into a larger theoretical organization that provides a model of human cognition. Some models, called structure-function models, are based on brain structure, centering functions in certain brain areas such as the frontal lobe (for locations and descriptions of brain areas, see diagram, p. 137). Other models, called process-oriented approaches, seek to categorize cognitive functions according to similarities in their component operations, providing functional mechanistic explanations of cognition. (For further discussion of these types of models, see the article by Evert and Oscar-Berman, pp. 89–96.) One such model defines alcoholics’ cognitive deficits by dividing functions such as memory into automatic, or stimulus-driven, component operations and reflective, or control, operations.

### Automatic-Reflective Operations Model

According to the automatic-reflective operations model, the brain carries out automatic operations fairly rapidly and in a manner not subject to much alteration. These operations use specialized (in contrast to general and flexible) brain circuits. Automatic operations tend to be reactions rather than planned actions. Usually, they are less complex than reflective operations, but they may play important roles in behaviors such as memory without awareness (i.e., implicit memory) and many facets of attention (i.e., those that are not subject to voluntary control). The model also proposes that the type of information gathered by automatic operations is driven by features of the external stimuli that initiate the operations and is only later evaluated and organized by the brain.

In contrast, reflective operations tend to be planned, voluntary actions. They generally are slower than automatic operations and involve the simultaneous use of several specialized processing systems (i.e., those involved in encoding, organizing, and evaluating information) as well as general systems in the brain. Reflective operations employ complex, multicomponent neural systems and rely on feedback loops in the brain that track and evaluate ongoing information processing. Reflective operations include monitoring and evaluating cognitive performance, planning and allocating cognitive resources, inhibiting and selecting responses, and transforming and integrating different forms of information. For example, a person uses reflective functions when he or she alters a behavior in response to a negative consequence (Cummings 1993; Shallice 1982; Stuss and Benson 1986).

### Alcoholics’ Deficits in Reflective Operations

Studies applying the automatic-reflective operations model have shown that tests of implicit memory, which is a relatively automatic process (wherein alcoholics perform tasks without being asked to recall how they learn to perform them), reveal no cognitive deficits among alcoholics (Weingartner et al. in press *a*). However, alcoholics do exhibit impairments in certain reflective components of memory. Specifically, alcoholics have difficulty performing such tasks as identifying the source of remembered information, inhibiting inappropriate responses in memory, and strategically allocating cognitive resources (Weingartner et al. in press *b*).

One test detects impairments in explicit, or voluntarily recalled, memory and therefore tests reflective operations of memory. The test involves presenting lists of words to subjects and asking them to propose associated words. After 2 days, the subjects are asked to remember the cue words and the words they selected. Although alcoholics appear to remember accurately the words from the previous session, they make more errors than do nonalcoholic subjects in identifying whether any given word was a cue provided by the tester or a word they generated themselves. Likewise, alcoholics make intrusion errors; that is, during similar tests of memory, they remember words that never were on the lists (Weingartner et al. in press *a*,*b*).

These findings show that at least some alcoholics have impairments in reflective cognitive functions, such as the ability to inhibit behavior and to monitor and evaluate ongoing behaviors. Furthermore, these functions may be impaired before people begin to drink alcohol abusively, implying that the functions may be involved in the development of abusive drinking (discussed below; Weingartner et al. in press *b*).^3^

Other “islands” of impaired and unimpaired functions have been identified in alcoholics. They have trouble planning and using cognitive strategies to organize their thoughts and memories, but if information is structured for them, alcoholics have no difficulty using that organization in processing the information. Alcoholics also appear more susceptible than nonalcoholics to having their actions influenced by surrounding stimuli. They tend to react rather than act voluntarily, and, as discussed below, this observation may have implications both for treatment and for detecting people at risk for developing alcoholism.

### Other Models

Some studies have compared cognitive performance in alcoholics with that seen in other populations. Based on a model proposing that alcoholism accelerates normal aging in the brain, some research has compared alcoholics of all ages with aging nonalcoholic adults. However, alcoholism does not appear to produce the same behavioral or neuropathological changes that normal aging generates.

Alcoholics’ cognitive profiles also differ from those seen in populations of patients with other neuropsychiatric disorders. Cognitive changes among alcoholics are unlike those seen in people with depression, anxiety, or dementia. Alterations seen in alcoholics’ cognitive functioning, however, may be similar to those of patients recovering from cocaine addiction. This resemblance implies that alcoholics’ impairments may provide a model of deficits that are concomitant with any substance addiction.

## Implications for Further Research

Because alcoholics are particularly susceptible to the influences of environmental stimuli, as described above, a setting, such as a bar, may have the power to sway a recovering alcoholic’s decision against remaining abstinent. Such stimuli also could contribute to factors influencing a person born with this cognitive susceptibility (i.e., someone who tends to react to stimuli rather than act voluntarily) to embark on an abusive drinking career or develop another form of psychopathology. Thus, alcoholics’ lack of control functions and their consequent reliance on reactions to stimuli have implications not only for their recovery but also for placing them at possible risk for developing alcoholism. Further studies of alcoholics’ cognitive impairments may uncover more factors that clinicians and other care givers could use to identify people at risk for developing the disease.

As studies describe an increasingly accurate picture of cognitive functioning in alcoholics, the etiology of alcoholism can be better understood. For example, the importance of cognitive risk factors in developing alcoholism can be assessed. This may in turn point to the role a person’s genetic predisposition plays in the disease (e.g., cognitive risk factors could contribute to an alcoholic predisposition). Studies that compare alcoholics’ cognitive profiles with those of people addicted to other drugs also will provide a more thorough understanding of addiction itself. Cognitive similarities or differences in the subjects, for instance, may help to reveal whether some addictions are actually attempts to alleviate psychiatric symptoms (e.g., depression or anxiety).

### Treatment

The results of cognitive neuroscientific research on alcoholism also has many implications for treatment. Although a general pattern of cognitive deficits among alcoholics has begun to emerge from these studies, variations exist in this pattern from person to person. Is there some way of differentiating alcoholic subgroups according to cognitive characteristics? Detailed profiles of this nature could help care givers match patients to the treatment methods most suited to their cognitive capacities. In addition, alcoholics’ stimulus-driven tendencies reinforce the idea that successful treatment programs must help to structure alcoholics’ lives. The treatments must anticipate scenarios and settings wherein recovering alcoholics are likely to resume drinking and perhaps teach them the skills needed to avoid or cope with such situations.

Future treatment research could explore ways to teach or strengthen control (i.e., reflective) operations in alcoholics as part of therapy. (For further discussion of strengthening cognitive functioning in alcoholics, see the article by Goldman, pp. 148–154.) No one knows if this type of training is possible, given that control operations, like computers, include both “hardware” systems and learned “programs” used to activate those systems. The hardware components of control functions probably cannot be changed substantially with therapy. If, however, alcoholics do not know how to rely on their control function programs, then minor programming adjustments through therapy might provide the tools necessary to bring them “online” again.

### Normal Human Cognition

Cognitive neuroscience’s work in the field of alcoholism has the potential to add to our knowledge of normal human cognition. The alcoholic’s pattern of spared and impaired operations demonstrates that these operations are distinct from one another in the brain, because they are affected by or impervious to different destructive agents, such as alcohol. Neuroscientists can conduct research that categorizes operations on the basis of their susceptibility to damage and further defines their distinctions, bringing the field nearer to its goal of discovering the unique features of each cognitive domain.

## Footnotes

1 Alcoholism is defined in this article using the definition given in the *Eighth Special Report to the U.S. Congress on Alcohol and Health*. According to this definition, alcoholism is “a disease characterized by abnormal alcohol-seeking behavior that leads to impaired control over drinking” (p. xxi).

2 Debate still exists as to whether cognitive changes are part of “normal” aging. However, changes in cognitive capacity and the ability to sustain cognitive effort and suppress inappropriate responses are thought by many researchers to occur as part of the normal aging process. For further information, see Light 1991.

3 The possibility that cognitive susceptibility to alcoholism may be inherited is part of a large and ongoing field of research into the heritability of many cognitive functions. Scientists are exploring both the behavioral and neurobiological aspects of inheriting a susceptibility to develop alcoholism. For further discussions of possible inherited cognitive predictors of alcoholism, see the article by Porjesz and Begleiter, pp. 108–112. See also Cloninger et al. 1981; Pihl et al. 1990; and Roy et al. 1987.
